# Objectifying Acupuncture Effects by Lung Function and Numeric Rating Scale in Patients Undergoing Heart Surgery

**DOI:** 10.1155/2013/219817

**Published:** 2013-03-14

**Authors:** Anna Maimer, Andrew Remppis, Falk-Udo Sack, Stefanie Ringes-Lichtenberg, Tobias Greten, Frank Brazkiewicz, Sven Schröder, Mario Goncalves, Thomas Efferth, Henry Johannes Greten

**Affiliations:** ^1^Department of Endocrinology and Metabolism, University of Heidelberg, Im Neuenheimer Feld 410, 69120 Heidelberg, Germany; ^2^Heidelberg School of Chinese Medicine, Karlsruher Straße 12, 69126 Heidelberg, Germany; ^3^ICBAS, University of Porto, Rua de Jorge Viterbo Ferreira No. 228, 4050-313 Porto, Portugal; ^4^Department of Cardiology, University of Heidelberg, Im Neuenheimer Feld 410, 69120 Heidelberg, Germany; ^5^Department of Heart Surgery, University of Heidelberg, Im Neuenheimer Feld 400, 69120 Heidelberg, Germany; ^6^HanseMerkur Center for Traditional Chinese Medicine, University Medical Center Hamburg-Eppendorf, Martinistraße 52, 20246 Hamburg, Germany; ^7^Department of Pharmaceutical Biology, Institute of Pharmacy and Biochemistry, Johannes Gutenberg University of Mainz, Staudinger Weg 5, 55099 Mainz, Germany

## Abstract

*Rationale*. Poststernotomy pain and impaired breathing are common clinical problems in early postoperative care following heart surgery. Insufficiently treated pain increases the risk of pulmonary complications. High-dose opioids are used for pain management, but they may cause side effects such as respiratory depression. *Study Design*. We performed a prospective, randomized, controlled, observer-blinded, three-armed clinical trial with 100 patients. Group 1 (*n* = 33) and Group 2 (*n* = 34) received one 20 min session of standardized acupuncture treatment with two different sets of acupoints. Group 3 (*n* = 33) served as standard analgesia control without additional intervention. Results. Primary endpoint analysis revealed a statistically significant analgesic effect for both acupuncture treatments. Group 1 showed a mean percentile pain reduction (PPR) of 18% (SD 19, *P* < 0.001). Group 2 yielded a mean PPR of 71% (SD 13, *P* < 0.001). In Group 1, acupuncture resulted in a mean forced vital capacity (FVC) increase of 30 cm^3^ (SD 73) without statistical significance (*P* = 0.303). In Group 2, posttreatment FVC showed a significant increase of 306 cm^3^ (SD 215, *P* < 0.001). *Conclusion*. Acupuncture revealed specific analgesic effects after sternotomy. Objective measurement of poststernotomy pain via lung function test was possible.

## 1. Introduction

Median sternotomy represents a surgical technique, in which the sternum is longitudinally incised and opened to get access to the organs of the mediastinum, for example, for heart transplantation, to set bypasses or valves on aortocoronary arteries, or to remove thymus tumors. Poststernotomy pain and impaired spontaneous breathing are common clinical problems in early postoperative care following heart surgery [1, 2]. Insufficiently treated pain impedes coughing and physiological rib cage expansion and, thus, increases the risk of pulmonary complications such as atelectasis and pneumonia [2]. 

High-dose application of opioids represents the standard therapy after sternotomy. It is controversially discussed whether opioids after median sternotomy lead to side effects such as respiratory depression, vomiting, nausea, and sedation [3, 4]. Therefore, combination therapy regimens have been applied containing opioids and traditional nonsteroidal anti-inflammatory drugs (tNSAIDs), cyclooxygenase-2 (COX-2) inhibitors, alpha2-inhibitors, or other nonopioid drugs such as paracetamol. The effectiveness of these combinations in pain reduction is, however, critically discussed [5–7]. Therefore, there is an urgent need for the improvement of pain therapy after median sternotomy. It has been suggested that electroacupuncture might be a valuable adjunct in managing poststernotomy pain [8, 9]. Whether needle acupuncture is a considerable asset in pain treatment after sternotomy is unknown as yet. 

The aim of the present investigation was, therefore, to investigate the effect of needle acupuncture on pain-induced reduction of lung function after median sternotomy. To this end, a prospective, randomized, controlled, patient- and observer-blinded clinical trial was performed with patients undergoing median sternotomy. 

## 2. Methods

The study was designed as a prospective, randomized, controlled, patient- and observer-blinded, three-armed clinical trail. Between September 2005 and August 2008, 100 thoracotomized patients were recruited from the intermediate intensive care unit (IMC) in the Department of Heart Surgery at Heidelberg University Hospital. Informed consent was obtained before subject enrollment according to a clinical trial protocol approved by the local Ethical Committee. 

### 2.1. Inclusion and Exclusion Criteria

Patients were included in the study if they had undergone conventional on-bypass surgery via median sternotomy and reported pain during deep inspiration with an intensity of at least 3 on a 1–10 numeric rating scale under standard analgesia with tNSARs and high-dose opioids. Patients had to be extubated and lucid. Patients meeting these criteria were excluded if they had a medical history of severe obstructive or restrictive pulmonary disease. Due to safety reasons, patients with a blood clotting time exceeding 4 min and/or a platelet count below 50.000 nL under anticoagulative therapy were also not accepted to the study. Additional ineligibility factors included acute general infection (fever, highly elevated leucocytes, or CRP) and/or local infection at the sites of potential needle insertion. Measures specifically influencing pain perception and/or lung function (i.e., inadvertent or accidental application of analgetics, physical exercise, physiotherapy treatment, and breathing exercises) were prohibited during the active 40 min study period (10 min screening and randomization, 5 min baseline assessment, 20 min acupuncture/observation, and 5 min control assessment) and would result in an exclusion from the study. Furthermore, intolerance to acupuncture treatment or sudden worsening of the physical condition would lead to a discontinuation of the study.

### 2.2. Randomization to Study Groups

Subjects were randomized to 3 study groups: Group 1 and 2 received one single 20 min session of standardized verum acupuncture treatment in addition to standard analgesia. Acupuncture was performed on 12 acupoints using sterile 0.25 × 40 mm surgical stainless steel needles. Needles were stimulated by rotation for approximately 5 sec during insertion, after 10 min, and before removal after 20 min. Additionally, in both groups, six of the selected 12 acupoints were treated with “blood-letting” or “leopard spotting technique” using sterile 033 mm 29-gauge needles. To rule out systematic acupuncturist-dependent fluctuations in overall treatment effects, all acupuncture treatments were carried out by the same physician, who had advanced training in traditional Chinese medicine (TCM).

The only systematic difference between acupuncture in Group 1 and Group 2 was the acupoint selection. In Group 2, the acupoint selection was based upon common classical Chinese diagnostic features shown by an a priori investigated representative patient collective (10 patients with varied sex, age, and cultural background with postoperative chest pain following sternotomy). A short summary of the acupoint selection is given in the following paragraph. Specific diagnostic technical terms are quoted and described with an auxiliary Western medical explanation in brackets. 

According to a well-known part of classical Chinese medical theory—the Shang Han Lun—which is systematized in the “Algor Laedens Theory” of the “Heidelberg model of TCM”, severe postoperative pain arises from “xue stasis” (relative hypoxis of a tissue arising from a disrupted microcirculation) due to “post-traumatic algor” (reduced microcirculation of a tissue following a trauma due to vasospasmic reflexes and immunological reactions) [10]. SP 10 was included as a classical acupoint to counteract xue stasis [11]. The common “orb pattern” (specific neuroaffective pattern with defined physical signs) presented by the patient collective indicated “a syndrome of the closing principle”—the simultaneous occurrence of a “splendor yang state” (neuroimmunological reaction with signs of the “stomach” and the “large intestine” orb pattern) and a “yin flectens state” (neuroimmunological reaction with signs of the “pericardium” and “liver” orb pattern) [10]. Respectively, ST 34, ST 44, PC 6, and LIV 2 were chosen as potent acupoints on the aforementioned orb meridians [11]. As all patients showed signs of a distinct “yin deficiency” (a labile neurovegetative regulation due to an overall deficiency of functional tissue), K 3 was added [11].

Acupoint selection in Group 1 served as a verum acupuncture control as it presented a form of inferior acupuncture—acupoints were exclusively selected according to their ascribed therapeutical actions and medical indications propagated by the acupuncture literature. LI 4 was included as a well-known, analgesic acupoint [11]. GV 20 and Ex 1 are generally ascribed psychovegetatively relaxing effects, possibly enhancing overall analgesia [11]. BL 60 is indicated to relief sharp chest pain and to loosen tension in the back muscles, GB 8 is recommended to treat feeling of pressure in the epigastric and thoracic regions, SI 6 is commonly used to uncramp the entire musculoskeletal system, and ST 8 is used to treat dyspnea [11].

A detailed list of the selected acupoints is illustrated in Tables 1 and 2. Group 3 served as a standard treatment control without additional acupuncture intervention. After baseline assessment, patients assigned to Group 3 were advised to avoid physical or mental stress and rest tranquilly in bed for 20 min under the supervision of the study investigator. In this way, Group 3 displayed the physiological fluctuations of pain perception and FVC over a 20 min interval without any medical intervention (observation). 

Randomization was stratified using consecutively numbered envelopes, containing the details of allocation. The envelopes were filled and sealed prior to study enrolment by an independent assistant according to a password-protected, computer-generated randomisation list. The envelopes were opened in a row by the acupuncturist at the bedside after recruitment. The designated observer and those patients allocated to an intervention arm (Group 1 or 2) were blind to treatment. Patients were informed that the study aimed to compare two different acupuncture treatments with respect to their analgesic effect after sternotomy.

### 2.3. Efficacy Parameters

Baseline and control efficacy assessment was carried out by a blinded observer and comprised two elements.

1. Dynamic pain assessment via numeric rating scale to record changes in subjective pain perception was carried out dynamically—during deep inspiration. Two different numeric rating scales were chosen; to semiquantify baseline chest pain, a conventional 1–10 NRS was used. To record the residual chest pain after treatment/observation in relation to the initial pain intensity, a 0%–100% NRS (subdivided into 10% sections) was implemented. The baseline pain intensity served as the individual “100%—reference pain” for this 0%–100% NRS.
2. Measurement of forced vital capacity (FVC) served as a physical and, thus, objective parameter to indicate analgesia-related functional improvements in rib cage expansion.

Percentile pain reduction (PPR) after treatment served as primary efficacy parameter, posttreatment change in FVC compared to baseline FVC was chosen as secondary efficacy parameter. For primary and secondary endpoint analyses, the Kruskal-Wallis test was performed as a 3-group global test, followed by the Wilcoxon test for post hoc analysis in case of a significant outcome. Correlation and subgroup analysis was performed to further investigate the a priori assumed pathophysiological connection between chest pain intensity and FVC measurement.

## 3. Results 

**Figure 1:**
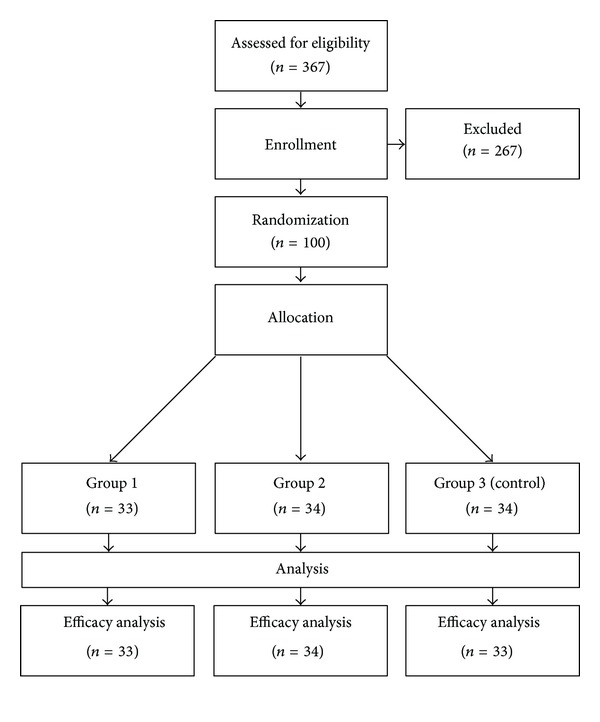
Participant flow.

Out of 367 screened patients, 100 patients met the inclusion criteria and were randomized to one out of three possible study groups. Participant flow through the trial is shown in Figure 1. Randomization resulted in an equal distribution of sociodemographic and clinical characteristics (see Table 3).

**Figure 2:**
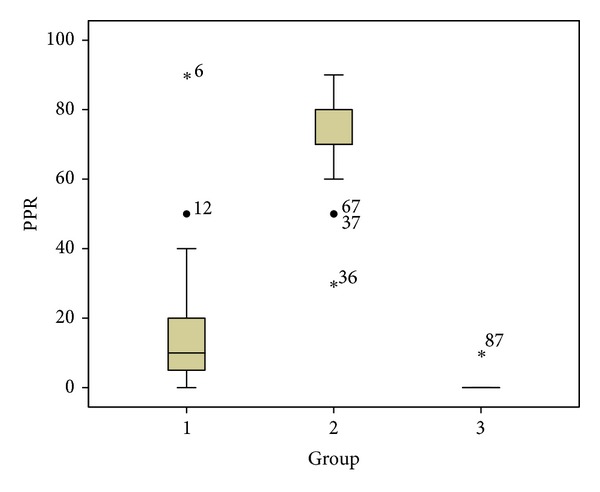
Distribution of PPR. Box-and-whisker plots represent lower quartile, median, upper quartile, maximum, minimum, outliers, and extreme values.

Primary endpoint analysis revealed a statistically significant analgesic effect for both acupuncture treatments—acupuncture in Group 1 resulted in a mean PPR of 18% (SD 19, *P* < 0.001), and acupuncture treatment in Group 2 yielded a mean PPR of 71% (SD 13, *P* < 0.001).  Figure 2 displays the distribution of PPR outcomes. 

**Figure 3:**
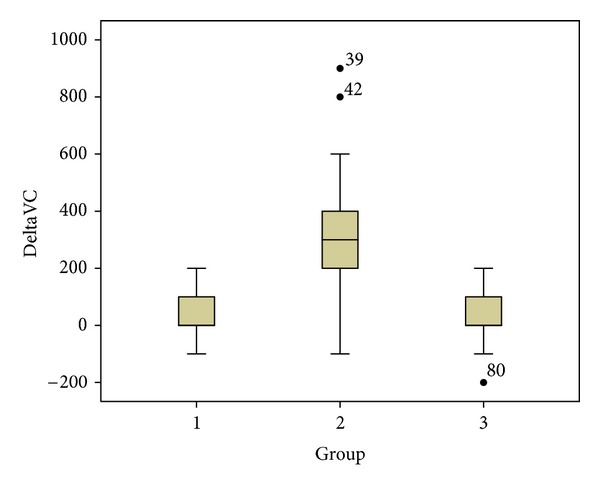
Distribution of FVC changes from baseline FVC. Box-and-whisker plots represent lower quartile, median, upper quartile, maximum, minimum, outliers, and extreme values.

**Figure 4:**
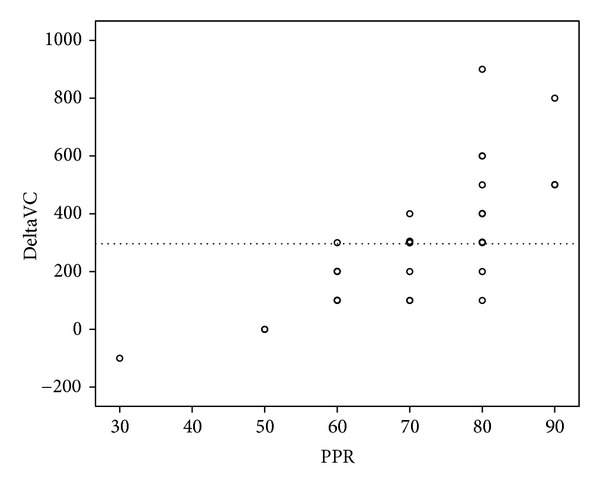
Correlation between PPR and posttreatment FVC changes in Group 2. Circles that are more intensely marked represent co-ordinates that occurred several times. The dotted line represents the lower limit for meaningful FVC changes (≥300 cm^3^).

In Group 1, acupuncture resulted in a mean FVC increase of 30 cm^3^ (SD 73), which failed to attain statistical significance (1 versus 3: *P*-value = 0.303). In Group 2, post-treatment FVC showed a statistically significant, average increase of 306 cm^3^ (SD 215, 2 versus 3: *P*-value < 0.001), with a strong correlation to PPR outcomes (spearman's rank correlation coefficient = 0.74). Pain reduction and FVC changes after acupuncture in Group 1 did not correlate in a statistically significant manner (*P* = 0.503).  Figure 3 illustrates the distribution of FVC-changes, and Figure 4 displays the correlation of PPR and FVC-changes in Group 2. 

**Figure 5:**
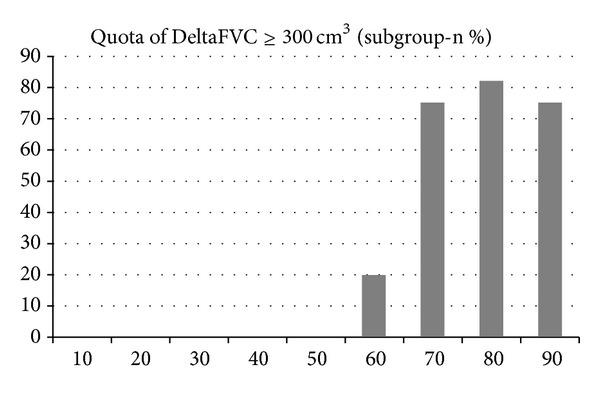
Subgroup analysis. Quota of clinically significant FVC increases (+≥300 cm^3^) in the individual PPR subgroups of Group 2.

Subgroup analysis revealed that meaningful FVC increases (≥300 cm^3^) exclusively occurred in patients with a PPR of 60%, or greater (see Figure 5). Below a PPR of 60%, the observed changes in FVC remained within the range of normal, acupuncture-independent fluctuations as shown by Group 3 patients.

## 4. Discussion

In the present investigation, we demonstrated statistically significant differences in immediate analgesic and functional effects between two types of acupuncture treatments compared to a control group in poststernotomy patients. The clinical trial showed that a lung function test can be used as an objective measurement of poststernotomy pain. Interestingly, only clinically significant analgesic effects (PPR ≥ 60%) led to functional improvements in lung function. This result further emphasizes the importance of maximal efficacy of postoperative analgesia to prevent pulmonary complications [2]. Some limitations of the present study may be discussed. The study period was short with no repeated acupuncture treatments, and the results only mirror immediate analgesic effects. Further studies may be performed in the future to address these issues. A Blinding of acupuncturists did not take place, because this is merely achievable. Furthermore, no placebo control was implemented in the study, since from an ethical point of view, a nontreatment group is not justifiable in patients suffering from severe pain. 

It is a long-lasting discussion in the scientific literature that strong postoperative pain and a considerable incidence of chronic pain after cardiac surgery and median sternotomy necessitate effective pain management [7]. A number of opioid-based treatment strategies have been described, including thoracic epidural anesthesia, spinal and intrathecal anesthesia, intercostal and paraventral blocks, or patient-controlled intravenous analgesia (PCS) [7]. In addition to combining opioids with tNSAIDs, COX-2 inhibitors, alpha2-inhibitors, or other drugs, several nonpharmacological strategies have been reported. Preoperative pain education of patients about postoperative pain resulted in less concerns about pain management [12]. Interestingly, electrical skin stimulation reduces pain perception of the organism. This technique was termed transcutaneous electrical nerve stimulation (TENS), which was successfully applied for post-sternotomy pain management in several randomized clinical trials [13–15]. The stimulation of skin for pain reduction indicates that acupuncture as a technique applied since ages to treat pain associated with many diseases and symptoms may also be helpful for poststernotomy pain management. Indeed, electroacupuncture has been reported to reduce poststernotomy pain and to improve pulmonary function [8, 9]. Electroacupuncture is based on the insertion of needles at specific acupoints together with electric current. 

In the present investigation, we clearly demonstrated that classical needle acupuncture without electrostimulation also led to significant pain reduction and lung function improvement. This may have impact on future concepts of pain management after median sternotomy in heart surgery. The fact that the acupuncture treatment of Groups 1 and 2 differ in their analgesic effects in the present study indicates that it considerably matters which acupoints are used and contradicts the view that acupuncture might only mediate nonspecific skin stimulation, which may or may not exceed placebo effects [16]. As imaging studies have delivered evidence of acupoint-specific functional magnetic resonance imaging patterns [17, 18] and the selection of acupoints presented the only systematic difference between acupuncture treatment in Groups 1 and 2, the observable difference in analgesic effects can be attributed to the summation effect of all twelve individual acupoint-specific reactions. Furthermore, it can be argued that the diagnosis-dependent selection of acupoints has significantly contributed to the analgesic superiority observed in Group 2. If acupoints are understood as reflex points that elicit specific neurovegetative alterations, it can be speculated that they might only yield satisfactory treatment results if they match the current vegetative status of a patient (which is expressed in the TCM diagnosis) [10]. However, it is also imaginable that psychological confounders may have distorted post-treatment pain assessment; minimal to moderate pain reductions—as frequently observed in Group 1—are possibly underrated if patients were disappointed by the acupuncture treatment effect, while patients, who were positively surprised by the analgesic effect of acupuncture treatment, might tend to overrate its effect. 

The exact mechanisms for acupuncture-mediated pain reduction in general and after median sternotomy are not well understood as yet. Different modes of action may account for pain reduction such as the release of endogenous opioids [7], adenosine A1 receptor-mediated antinociceptive effects [19], mast cell degranulation, and and the release of substance *P*, amongst others [20–26]. 

In conclusion, the present clinical trial demonstrated that acupuncture revealed specific effects in pain management after median sternotomy. Objective measurement of poststernotomy pain by a lung function test was possible and enabled to distinguish between functionally insignificant pain reduction (resulting in no improvement of breathing) and clinically significant analgesia effects of acupuncture. 

## Figures and Tables

**Table 1:** Acupoint selection in Group 1 (control acupuncture).

Engl. abbr.	Latin abbr.	Latin notation	Chinese notation	Needling technique	Localisation
LI 4	IC 4	Valles coniunctae	Hegu	blood-letting	bilateral
SI 6	IT 6	Senectus felix	Yanglao	blood-letting	bilateral
BL 60	V 60	Olympus	Kunlun	blood-letting	bilateral
Ex 1	Ex 1	Atrium impressionis	Yintang	conventional needle insertion	median
GV 20	Rg 20	Conventus omnium	Baihui	conventional needle insertion	median
GB 8	F 8	Apex auriculi	Erdian/Shuanijue	conventional needle insertion	bilateral
ST 8	S 8	Retinens capitis	Touwei	conventional needle insertion	bilateral

**Table 2:** Acupoint selection in Group 2 (classical Chinese acupuncture).

Engl. abbr.	Latin abbr.	Latin notation	Chinese notation	Needling technique	Localisation
PC 6	PC 6	Clusa interna	Neiguan	blood-letting	bilateral
ST 34	S 34	Monticuli septi	Liangqiu	blood-letting	bilateral
SP 10	L 10	Mare xue	Xuehai	conventional needle insertion	bilateral
ST 44	S 44	Vestibulum internum	Neiting	blood-letting	bilateral
K 3	R 3	Rivulus major	Taixi	conventional needle insertion	bilateral
LIV 2	H 2	Interstitium ambulatorium	Xingjian	conventional needle insertion	bilateral

**Table 3:** Patients' characteristics at baseline.

	Group 1	Group 2	Group 3 (control)	Total	*P* value
*N*	33	34	33	100	
Male gender*	24 (73%)	22 (65%)	26 (79%)	72 (72%)	0.436^∞^
Age (years)					0.295^#^
Mean (SD)	65 (10)	68 (11)	66 (10)	66 (10)	
BMI (kg/m^2^)					0.515^#^
Mean (SD)	28 (4)	29 (5)	29 (4)	28 (4)	
Operation type					
On-bypass*	33 (100%)	34 (100%)	33 (100%)	100 (100%)	
Coronary revascularisation*	17 (51%)	20 (58%)	21 (63%)	58 (58%)	
Valve reconstruction or replacement*	15 (45%)	14 (41%)	15 (45%)	44 (44%)	
Aortic reconstruction after dissection*	1 (3%)	0 (0%)	0 (%)	1 (1%)	
Chest pain baseline (0–10 NRS)					
Median (IQR)	6 (2)	5 (2)	5 (2)	5 (2)	0.653^#^
Mean (SD)	5.5 (1.2)	5.2 (1.4)	5.3 (1.3)	5.3 (1.3)	
FVC baseline (cm^3^)					
Median (IQR)	1200 (600)	1000 (550)	1200 (400)	1150 (600)	0.082^#^
Mean (SD)	1294 (426)	1147 (405)	1275 (325)	1238 (390)	

*The numbers in parentheses display the percentage for dichotomous variables.

^#^ *P* values were calculated using the Kruskal-Wallis test.

^∞^ *P* values were calculated using the *χ* ^2^-test.

Abbreviations—SD: standard deviation; IQR: interquartile range.
